# Comparison of mitral valve repair vs. replacement for mitral valve regurgitation

**DOI:** 10.1093/ehjqcco/qcae108

**Published:** 2025-01-07

**Authors:** Maciej Dębski, Syed Qadri, U Bhalraam, Karolina Dębska, Vassilios Vassiliou, Joseph Zacharias

**Affiliations:** Lancashire Cardiac Centre, Blackpool Teaching Hospitals NHS Foundation Trust, Whinney Heys Rd, Blackpool FY3 8NP, UK; Norwich Medical School, University of East Anglia, Norwich NR4 7TJ, UK; Department of Cardiology, Norfolk and Norwich University Hospital, Colney Ln, Norwich NR4 7UY, UK; Lancashire Cardiac Centre, Blackpool Teaching Hospitals NHS Foundation Trust, Whinney Heys Rd, Blackpool FY3 8NP, UK; Norwich Medical School, University of East Anglia, Norwich NR4 7TJ, UK; Department of Cardiology, Norfolk and Norwich University Hospital, Colney Ln, Norwich NR4 7UY, UK; Department of Cardiology, Norfolk and Norwich University Hospital, Colney Ln, Norwich NR4 7UY, UK; Norwich Medical School, University of East Anglia, Norwich NR4 7TJ, UK; Department of Cardiology, Norfolk and Norwich University Hospital, Colney Ln, Norwich NR4 7UY, UK; Lancashire Cardiac Centre, Blackpool Teaching Hospitals NHS Foundation Trust, Whinney Heys Rd, Blackpool FY3 8NP, UK

**Keywords:** Mitral regurgitation, Mitral valve repair, Mitral valve replacement

## Abstract

**Background:**

Mitral regurgitation (MR) is a prevalent valvular abnormality categorized as primary or secondary based on aetiology. Surgical intervention, particularly mitral valve repair, is often preferred over replacement due to its association with better outcomes. However, the benefits of repair vs. replacement, especially in secondary MR, remain debated.

**Objectives:**

This study aims to evaluate the long-term survival and reoperation rates in patients undergoing mitral valve repair compared to mitral valve replacement for MR in a cardiothoracic surgery unit in North-West England and in subgroups with degenerative and secondary aetiology.

**Methods and results:**

We analysed 1724 eligible patients undergoing first-time mitral valve surgery (repair: *n* = 1243; replacement: *n* = 481) between 2000 and 2021. The primary outcome was all-cause mortality. Genetic matching and overlap weighting were used to balance baseline characteristics. Median follow-up was 7.1 years. In the matched cohort, mitral valve replacement was associated with higher rates of blood transfusion (29% vs. 22%), longer Intensive Care Unit (ICU) stays, and more strokes (3.7% vs. 0.4%). While 90-day mortality did not differ significantly between groups, long-term follow-up showed a survival advantage for repair [Hazard ratio: 1.32, 95% confidence interval: 1.08–1.63]. Although repair had higher reoperation rates (4.3% vs. 2.1%), the composite of death or reoperation did not differ significantly. In the degenerative MR subgroup, repair showed superior long-term survival, whereas in secondary MR, no significant survival difference was observed between strategies.

**Conclusion:**

Among patients suitable for either surgical strategy, mitral valve repair showed better long-term survival compared to replacement, particularly in degenerative MR. However, this advantage was not observed in secondary MR.

Key Learning Points **What is already known**

- Both mitral valve repair and replacement have their roles in the management of mitral valve disease.
- The choice between the two depends on various factors, including the underlying cause of the valve dysfunction, the patient's age and comorbidities, and the surgeon's expertise.
- Observational studies in degenerative mitral valve disease have shown that mitral valve repair is associated with better long-term survival rates compared to replacement; however, is less durable. Randomized controlled trial in severe ischaemic mitral regurgitation did not show survival benefit with repair.

**What this study adds**

- In patients meeting study criteria who underwent first-time mitral valve surgery with median follow-up of 7.1 years, repair was associated with better long-term survival in matched analyses, particularly in degenerative disease, while showing no survival advantage in secondary mitral regurgitation.
- Using robust statistical methodology with genetic matching and overlap weighting, this study demonstrates that mitral valve replacement is associated with higher rates of blood transfusion, cerebrovascular complications, and longer hospital stays, even after balancing baseline characteristics.
- This study broadens current understanding of early post-operative outcomes by identifying specific complications more common with replacement, particularly highlighting the increased risk of stroke, which may influence surgical decision-making.

## Introduction

Mitral regurgitation (MR) is the second most common valvular abnormality worldwide, affecting over 2% of the total population and has a prevalence that increases with age.^1–3^ MR is categorized as primary or secondary according to the underlying mechanism. Primary MR results from structural abnormalities of the valve apparatus (degenerative, rheumatic, or infective processes), while secondary MR occurs due to left ventricular dysfunction from ischaemic or non-ischaemic cardiomyopathies, or geometric changes from long-standing atrial fibrillation leading to annular dilatation.

Surgical management of the mitral valve depends largely on the valve anatomy, which is assessed mainly by echocardiography and intraoperative inspection. This assessment helps determine whether a durable repair can be performed or if valve replacement is necessary. The optimal surgical approach remains a subject of ongoing discussion, particularly given the diversity of underlying pathologies. According to ACC/AHA, ESC/EACTS, and NICE UK Guidelines for the Management of Valvular Heart Diseases, when surgery is considered for primary mitral valve disease, if mitral valve repair is expected to be durable according to the Heart Team evaluation, it is preferred over mitral valve replacement as it is associated with better survival compared to mitral valve replacement.^4–6^ Repair has been associated with lower operative mortality, greater longevity, a reduced risk of endocarditis, and better quality of life as compared with mitral valve replacement. When repair is not feasible, experts advocate for mitral valve replacement with preservation of the subvalvular apparatus. These guideline recommendations for primary mitral valve disease are based solely on observational studies. However, in secondary mitral valve regurgitation, the advantage of repair is less clear. The randomized controlled trial conducted by Cardiothoracic Surgical Trials Network reported no difference in left ventricular end-systolic volume index or survival at 2 years between patients with severe ischaemic MR randomized to repair or replacement. Moreover, replacement provided more durable correction of MR and fewer cardiovascular readmissions.^7^

Our aim was to evaluate long-term survival and reoperation rates in patients undergoing first-time mitral valve surgery for MR at a high-volume UK centre with expertise in both conventional and minimally invasive techniques, with particular focus on outcomes in different pathological subgroups.

## Methods

### Ethics statement

The study was approved by the Research Ethics Committee—Health Research Authority, and in line with other retrospective studies, the need for informed consent was waived. The database was anonymized before analysis.

### Study population and study design

We conducted a longitudinal, observational, retrospective cohort study in Lancashire Cardiac Centre of all consecutive patients undergoing mitral valve surgery for MR between January 2000 and December 2021 who met the following criteria: first mitral valve surgery, either via thoracoscopically guided right minithoracotomy (MIMVS) or via median sternotomy, with or without concomitant coronary artery bypass graft surgery (CABG), tricuspid valve surgery (TVS), adjunctive cardiac procedures for atrial fibrillation (AF), and patent foramen ovale closure. Patients with any previous valvular surgery, previous percutaneous transvenous mitral commissurotomy, simultaneous aortic valve repair/replacement or surgery on ascending aorta, mixed mitral valve disease with moderate or severe mitral stenosis or more than mild mitral valve stenosis, and those younger than 18 were excluded. Demographic and pre-operative information, operative data, and in-hospital post-operative outcomes for all patients were retrieved from the prospectively maintained institutional database validated for the purpose of outcome reporting to The National Adult Cardiac Surgery Audit conducted by the National Institute for Cardiovascular Outcomes Research.

### Definitions

Pre-operative co-morbidities were defined according to the definitions used in the EuroSCORE risk stratification model.^8^ Incisions included sternotomy and mini-thoracotomy approaches. MIMVS and conventional sternotomy access have been described previously.^9^^,^^10^ The choice of incision was dependent on surgeon preference. MR aetiology was categorized as degenerative, ischaemic/functional, rheumatic, infective endocarditis, or congenital. Degenerative disease was defined as single- or multi-segment prolapse due to chordal elongation or rupture. Rheumatic valve disease was defined as reduced leaflet motion in systole and diastole associated with leaflet thickening and commissural fusion. Endocarditis was defined by the evidence of acute or chronic vegetations or leaflet perforation, or a history of endocarditis. Secondary MR included ischaemic aetiology caused by papillary muscle displacement posteriorly as a result of asymmetrical left ventricular dilatation due to ischaemic cardiomyopathy or functional MR due to other non-ischaemic pathologies such as dilated cardiomyopathy, hypertrophic cardiomyopathy, or left atrial dilation and in the absence of structural mitral valve disease.

### Intervention

Patients with mitral valve repair (with or without annuloplasty) were compared with those who underwent mitral valve replacement (with bioprosthetic or mechanical valve). Decisions regarding repair or replacement as well as prosthesis choice were made according to surgeon preference. Repair was favoured in degenerative MR wherever technically possible.^10^^,^^11^ Surgery via sternotomy was performed on cardiopulmonary bypass using cold crystalloid cardioplegic arrest with bi-caval cannulation. Exposure was usually achieved via the Waterston's incision or via a bi-atrial vertical trans-septal incision. Moderate hypothermia of 32–34°C was used in the majority of the patients. Port access surgery was done with femoral bypass and right lateral mini-thoracotomy/port access with 3D video assisted thoracoscope. The patients who had mitral valve repair had valve and subvalvular apparatus evaluated. If the chordal apparatus was intact, they were offered simple ring annuloplasty. In the case of chordal rupture, artificial chords were used to repair and resuspend the prolapsing segments of the leaflets along with ring annuloplasty. We used mostly semi-rigid complete annuloplasty rings. When the mitral valve replacement was offered, care was given to preserve sub valvular apparatus including chordae tendineae and papillary muscles. The interrupted sutures were used to attach the prosthetic valve to the mitral valve annulus using 2.0 Ethibond pledgeted sutures.^10^^,^^11^

### Outcomes

The primary outcome measure was time to all-cause mortality. Secondary endpoints were 90-day all-cause mortality and composite of time to first mitral valve reoperation and all-cause mortality. Data on reoperations were available only if performed in our centre. Any patient who died on the day of surgery was assigned a nominal survival and post-operative length of stay of 0.5 days. Information on vital status and date of death was obtained from the UK's Office for National Statistics through the end of February 2023.

### Statistical analysis

Statistical analyses were performed using R 4.3.1 (R Core Team, Vienna, Austria). A *P* value of 0.05 was considered statistically significant. Tests were two tailed. Continuous variables are presented as mean [standard deviation (SD)] in balance tables and median [interquartile range (IQR)] in outcomes tables and were compared using the weighted Mann–Whitney *U* test. Categorical variables are presented as counts and percentages and compared with weighted Pearson's chi-square test. Multiple imputation by chained equations was done using the mice package v3.13.0 and imputed the missing data in the following variables: history of hypertension (2.5%; 43/1724 missing), history of neurological disease (1%), history of pulmonary disease (0.2%), previous cardiac surgery (0.6%), mitral valve pathology (0.4%), diabetes (0.1%), blood loss (0.4%), blood used (yes/no) (0.1%), cumulative bypass time (0.1%), and cumulative cross clamp time (0.1%).^12^

A univariable logistic regression analysis was first conducted for each potential predictor to assess its association with the type of mitral valve procedure (replacement vs. repair). Variables with *P*-values less than 0.05 were considered for inclusion in the multivariable logistic regression model. Multicollinearity was evaluated using the variance inflation factor (VIF), and variables with VIF greater than 5 were excluded to prevent multicollinearity. The final multivariable model was determined using a stepwise selection method based on the Akaike information criterion to achieve the most parsimonious model. Model performance was assessed by calculating the overall accuracy, sensitivity, specificity, positive predictive value, negative predictive value, and area under the receiver operating characteristic curve. The Hosmer–Lemeshow test was used to evaluate the goodness-of-fit of the logistic regression model. Cross-validation was performed using a 10-fold cross-validation method to estimate the model's predictive accuracy on unseen data.

To balance the cohorts, we used genetic matching with replacement using the MatchIt package in R,^13^ which calls functions from the Matching package to model a dichotomous outcome of replacement or repair.^14^^,^^15^ After comparing different matching approaches, genetic matching was selected as it achieved superior covariate balance compared to optimal matching or nearest neighbour matching as evidenced by lower standardized mean differences (SMDs) across covariates. The calliper width was set at 0.1 and the population size at 1000. The exact matching was performed on native mitral valve pathology (congenital, degenerative, infective endocarditis, ischaemic/functional, and rheumatic) and genetic matching was conducted on the following variables: binary covariates—New York Heart Association (NYHA) collapsed to classes I/II and III/IV, previous cardiac surgery, arterial hypertension, diabetes, chronic lung disease, history of cerebrovascular accident or transient ischaemic attack, unstable angina, critical perioperative state, pulmonary hypertension, AF, extracardiac arteriopathy, creatinine >200 µmol/L pre-operatively, concomitant CABG, TVS, nominal covariates—smoking status (never smoker, ex-smoker, current smoker), extent of coronary artery disease (CAD) (not investigated, no vessel with >50% diameter stenosis, one vessel with >50% diameter stenosis, two vessels with >50% diameter stenosis, three vessels with >50% diameter stenosis), recent myocardial infarction (within 90 days before operation), left ventricular ejection fraction category (LVEF) [good (LVEF >50%), moderate (LVEF 31–50%), poor (LVEF 30% or less)], operative priority (elective, urgent, or emergency/salvage); continuous covariates—age, body mass index, logistic EuroSCORE. In addition, we performed a separate genetic matching on degenerative MR patients only and ischaemic/functional patients only using the covariates listed above except extent of CAD and unstable angina and no exact matching, population size 10 000, and calliper 0.2 in degenerative MR subgroup and 0.15 in ischaemic/functional MR subgroup.

To verify the robustness of the results, we performed sensitivity analysis using overlap weighting (OW), a propensity score-based technique that emphasizes patients with characteristics making them eligible for either treatment strategy.^16^ Overlap weights are set to 1 at points of maximal overlap between propensity score functions, up-weighting patients with balanced characteristics while down-weighting those in the tails of the distribution. This approach offers advantages over traditional inverse probability weighting, including exact covariate balance and emphasis on medical equipoise. We used identical predictor variables as in genetic matching for both the overall cohort and subgroup analyses.

In both matched and OW analysis, we assessed the balance using absolute SMD and Kolmogorov–Smirnov (KS) statistic. A difference of 10% or less in SMD was considered to indicate a well-balanced result. KS statistics measure the greatest distance between the empirical cumulative distribution functions for each variable between two groups. The statistic is bounded at 0 and 1, with 0 indicating perfectly identical distributions and 1 indicating perfect separation between the distributions (i.e. no overlap at all); values close to 0 are thus indicative of balance. Covariate balance was assessed using cobalt package.^17^ As we performed genetic matching with replacement, when estimating the hazard ratios and 95% confidence intervals (CI) in time to event analyses, we accounted for control unit multiplicity (i.e. repeated use of subjects with MV repair) and within-pair correlations using Austin and Cafri custom variance estimator.^18^ Survival was depicted with the weighted Kaplan–Meier curves for genetic matched cohorts including weighted at risk tables and adjusted survival curves for overlap-weighted cohorts. The proportionality of hazard assumption was checked for all time-to-event analyses using Schoenfeld's Residuals global test.

## Results

### Baseline characteristics

**Figure 1:**
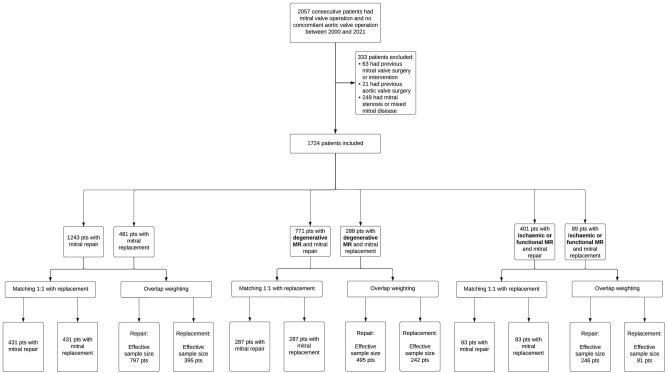
Study flow chart.

We analysed a total of 1724 consecutive patients with MR who met the study eligibility criteria (*Figure 1*). Among these, 1243 (72%) underwent mitral valve repair (1230 with annuloplasty ring and 13 without a ring), and 481 patients (28%) underwent mitral valve replacement (230 with a bioprosthetic valve and 251 with a mechanical valve). Median follow-up was 7.1 (IQR, 3.6–11.6) years and the total was 13 613 patient-years. Follow-up duration was similar between groups: 6.9 (IQR, 3.6–11) years in the repair group and 7.8 (IQR, 3.5–13.6) years in the replacement group. Baseline patient demographics, risk variables, and comorbidities are summarized in *Table 1*. There were 57 patients with previous cardiac surgery: 46 had previous CABG, six had congenital surgery (four atrial septal defect repair, one ventricular septal defect, and one coarctation) and five had other surgery such as myomectomy or atrial myxoma excision. Minimally invasive MV surgery was started in our centre in 2007 and was performed on 449 (26%) patients. Patients with concomitant tricuspid valve intervention had annuloplasty rings in all cases.

**Table 1:** Patient characteristics according to mitral valve repair or replacement in the overall population, genetic-matched cohorts, and overlap-weighted cohort

	All patients			Genetic matched			Overlap weighted^a^	
Characteristic	Repair, *N* = 1243	Replacement, *N* = 481	SMD	Repair, *N* = 431	Replacement, *N* = 431	SMD	Repair, ESS = 797	Replacement, ESS = 395
Age (years)	67 (12)	66 (12)	0.05	68 (11)	67 (12)	0.08	67 (12)	67 (12)
Female sex	431 (35%)	211 (44%)	0.19	181 (42%)	189 (44%)	0.04	40%	40%
Body mass index (kg/m^2^)	26.3 (4.6)	26.6 (5.1)	0.06	26.5 (4.1)	26.5 (4.9)	0.01	26 (5)	26 (5)
Smoking status								
Current smoker	76 (6.1%)	46 (9.6%)	0.13	28 (6.5%)	35 (8.1%)	0.06	8%	8%
Ex-smoker	586 (47%)	252 (52%)	0.11	238 (55%)	230 (53%)	0.04	51%	51%
Never smoked	581 (47%)	183 (38%)	0.18	165 (38%)	166 (39%)	0	41%	41%
Chronic pulmonary disease	152 (12%)	66 (14%)	0.04	49 (11%)	60 (14%)	0.07	13%	13%
Pulmonary hypertension	279 (22%)	202 (42%)	0.43	184 (43%)	176 (41%)	0.04	34%	34%
Previous CVA or TIA	122 (9.8%)	49 (10%)	0.01	38 (8.8%)	41 (9.5%)	0.02	10%	10%
Neurological dysfunction	39 (3.1%)	33 (6.9%)	0.17	29 (6.7%)	27 (6.3%)	0.02	5%	5%
Diabetes	121 (9.7%)	46 (9.6%)	0.01	38 (8.8%)	42 (9.7%)	0.03	10%	10%
Creatinine >200 µmol/L	59 (4.7%)	54 (11%)	0.24	41 (9.5%)	41 (9.5%)	0	8%	8%
Atrial fibrillation	699 (56%)	280 (58%)	0.04	259 (60%)	250 (58%)	0.04	57%	57%
Arterial hypertension	694 (56%)	242 (50%)	0.11	214 (50%)	223 (52%)	0.04	53%	53%
Previous cardiac surgery	34 (2.7%)	23 (4.8%)	0.11	20 (4.6%)	20 (4.6%)	0.01	3.5%	3.5%
NYHA class								
1/2	674 (54%)	195 (41%)		167 (39%)	180 (42%)		46%	46%
3/4	569 (46%)	286 (59%)	0.28	264 (61%)	251 (58%)	0.06	54%	54%
Left ventricular ejection fraction (LVEF) category								
Poor (LVEF ≤30%)	77 (6.2%)	41 (8.5%)	0.09	37 (8.6%)	35 (8.1%)	0.02	8.5%	8.5%
Moderate (LVEF 31–50%)	260 (21%)	115 (24%)	0.07	93 (22%)	100 (23%)	0.04	22%	22%
Good (LVEF >50%)	906 (73%)	325 (68%)	0.1	301 (70%)	296 (69%)	0.02	70%	70%
Extracardiac arteriopathy	74 (6.0%)	56 (12%)	0.2	35 (8.1%)	45 (10%)	0.07	9%	9%
Extent of coronary artery disease								
Not investigated	33 (2.7%)	12 (2.5%)	0.01	15 (3.5%)	9 (2.1%)	0.09	2.6%	2.6%
No vessel with >50% diameter stenosis	764 (61%)	234 (49%)	0.24	199 (46%)	211 (49%)	0.06	52%	52%
One vessel with >50% diameter stenosis	128 (10%)	77 (16%)	0.19	73 (17%)	72 (17%)	0.01	14%	14%
Two vessels with >50% diameter stenosis	95 (7.6%)	60 (12%)	0.14	46 (11%)	53 (12%)	0.05	10.5%	10.5%
Three vessels with >50% diameter stenosis	223 (18%)	98 (20%)	0.06	98 (23%)	86 (20%)	0.07	20.5%	20.5%
Myocardial infarction in the last 90 days	103 (8.3%)	53 (11%)	0.09	36 (8.4%)	45 (10%)	0.06	9%	9%
Unstable angina	24 (1.9%)	31 (6.4%)	0.23	18 (4.2%)	21 (4.9%)	0.03	4%	4%
Critical preoperative state	28 (2.3%)	53 (11%)	0.36	23 (5.3%)	28 (6.5%)	0.04	5%	5%
Logistic EuroSCORE	7 (8)	11 (13)	0.35	9 (10)	10 (10)	0.02	9 (10)	9 (10)
Native mitral pathology								
Congenital	10 (0.8%)	7 (1.5%)	0.06	3 (0.7%)	3 (0.7%)	0	1.3%	1.3%
Degenerative	771 (62%)	288 (60%)	0.05	279 (65%)	279 (65%)	0	64%	64%
Ischaemic/functional regurgitation	401 (32%)	89 (19%)	0.31	80 (19%)	80 (19%)	0	23%	23%
Infective endocarditis	47 (3.8%)	44 (9.1%)	0.2	30 (7.0%)	30 (7.0%)	0	7%	7%
Rheumatic	14 (1.1%)	53 (11%)	0.36	39 (9.0%)	39 (9.0%)	0	4%	4%
Operative urgency								
Elective	1053 (85%)	339 (70%)	0.35	338 (78%)	321 (74%)	0.09	78%	78%
Urgent	176 (14%)	105 (22%)	0.22	80 (19%)	91 (21%)	0.06	19%	19%
Emergency or salvage	14 (1.1%)	37 (7.7%)	0.36	13 (3.0%)	19 (4.4%)	0.05	32%	32%
Coronary artery bypass graft	413 (33%)	213 (44%)	0.23	201 (47%)	192 (45%)	0.04	42%	42%
Tricuspid valve surgery	309 (25%)	79 (16%)	0.21	59 (14%)	69 (16%)	0.06	18%	18%

Data as *n* (%) or mean (SD).

a Overlap weights provide exact covariate balance and all SMD are equal to 0.

In the unadjusted (all patients) cohort, patients who underwent mitral valve replacement were more likely to be female, have pulmonary hypertension, and present with neurological dysfunction compared to those who underwent repair. They also had a higher logistic EuroSCORE, indicating a higher predicted operative risk. Additionally, replacement patients were more likely to have extracardiac arteriopathy, unstable angina, undergo CABG, present with more advanced NYHA class, be in a critical pre-operative state, have an urgent or emergency/salvage operation, and less likely to undergo TVS.

### Characteristics affecting treatment choice

In the unmatched cohort, multivariable logistic regression identified the following characteristics independently associated with mitral valve replacement as compared to mitral valve repair: female sex, pulmonary hypertension, neurological dysfunction, arterial hypertension, previous cardiac surgery, NYHA class III/IV, extracardiac arteriopathy, LVEF <30% as compared to >50%, critical pre-operative state, infective endocarditis, rheumatic MR, urgent, or emergency/salvage operation as compared to elective and concomitant CABG. Ex-smokers had increased odds of replacement compared to those who never smoked, while current smokers showed a trend towards higher odds that did not reach statistical significance. On the other hand, arterial hypertension, intervention on tricuspid valve, and ischaemic/functional MR were associated with higher chances of undergoing mitral valve repair as compared to replacement (Supplementary material online, *Table S1*).

### Outcomes

**Figure 2:**
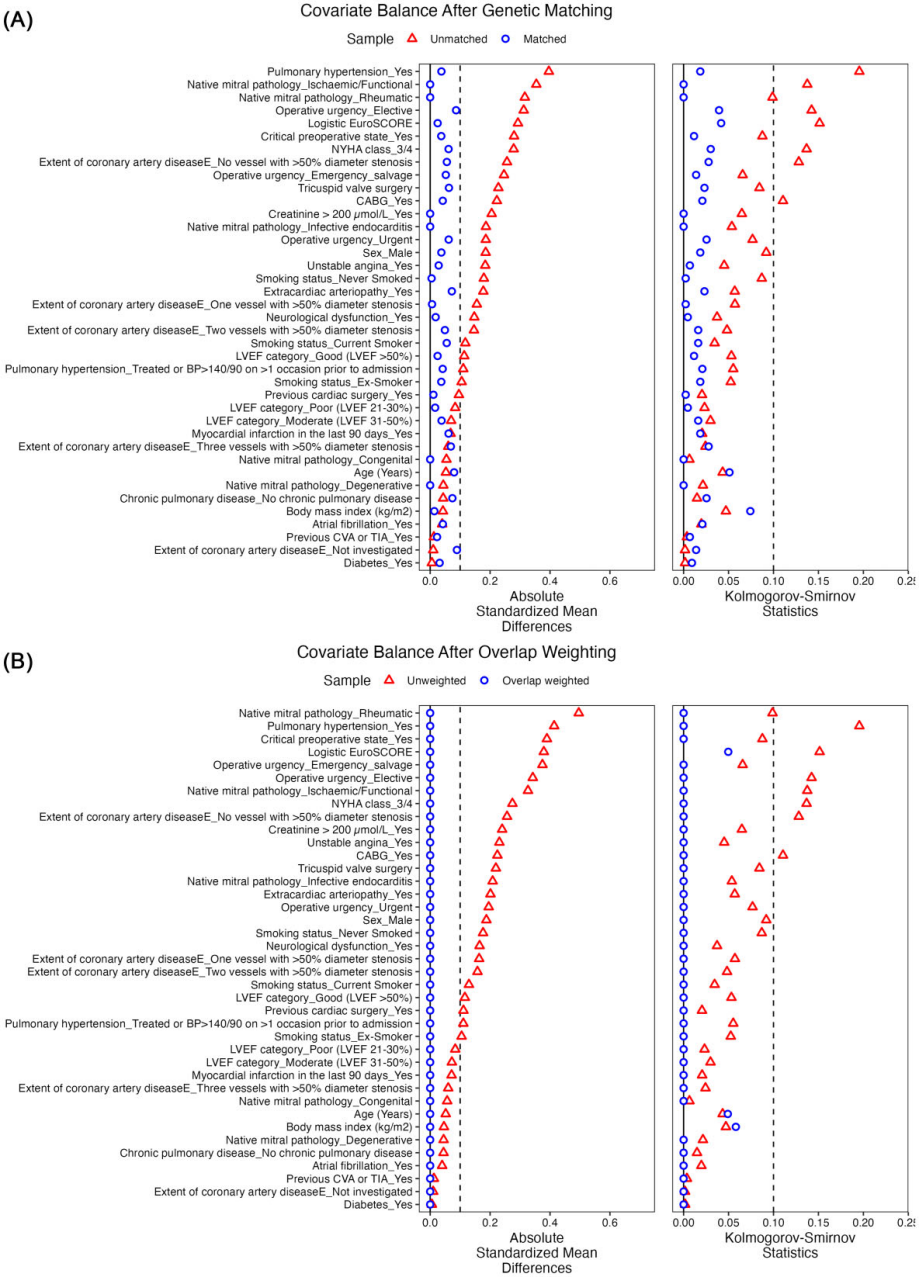
Love plot visually summarizing the covariate balance using absolute standardized mean difference and Kolmogorov–Smirnov statistic in the matched samples (*A*) and overlap weighted sample (*B*).

After applying genetic matching and overlap weighting methods, excellent balance was achieved between groups (*Table 1*, *Figure 2*). In the genetic-matched cohort, both groups had 431 patients each, with SMDs below 10% for all matching variables. Similarly, in the overlap-weighted cohort, exact covariate balance was achieved, with effective sample sizes of 797 for repair and 395 for replacement.

In the matched cohort, replacement patients had significantly higher blood loss, required more blood transfusions (29% vs. 22%, *P* = 0.023), and experienced longer intensive care unit and post-operative lengths of stay compared to repair patients (*Table 2*). The rate of stroke was notably higher in the replacement group (3.7% vs. 0.4%, *P* = 0.013). A higher proportion of replacement patients were discharged to other acute hospitals or non-acute care facilities rather than directly home. In the OW cohort, similar patterns were observed.

**Table 2:** Intraoperative and postoperative outcomes among patients with mitral valve repair and replacement in all patients, genetic-matched subset, and overlap-weighted cohort

	All patients			Genetic matched (weighted)			Overlap weighted		
Characteristic	Repair, *N* = 1243	Replacement, *N* = 481	*P*-value	Repair, *N* = 282	Replacement, *N* = 431	*P*-value	Repair, ESS = 797	Replacement, ESS = 395	*P*-value
Blood loss (mL)	400 (260–615)	440 (280–760)	<0.001	400 (260–620)	440 (300–780)	0.011	420 (280–645)	440 (300–800)	0.02
Blood used	233 (19%)	142 (30%)	<0.001	61 (22%)	124 (29%)	0.023	21%	29%	0.003
Transfused blood (units)	0 (0–0)	0 (0–1)	<0.001	0 (0–0)	0 (0–1)	0.012	0 (0–0)	0 (0–1)	0.002
Cumulative bypass time (min)	151 (127–181)	150 (116–185)	0.26	155 (129–186)	149 (116–184)	0.031	151 (126–180)	151 (117–186)	0.589
Cumulative cross clamp time (min)	106 (84–127)	98 (77–131)	0.035	108 (85–132)	98 (77–131)	0.016	106 (84–127)	99 (79–131)	0.309
Ventilation duration			<0.001			0.077			0.19
Immediate extubation	5 (0.4%)	3 (0.6%)		1 (0.4%)	2 (0.5%)		0.3%	0.7%	
<12 h	1066 (86%)	355 (74%)		238 (84%)	329 (76%)		83%	77%	
12–24 h	116 (9%)	69 (14%)		29 (10%)	61 (14%)		11%	13%	
>24 h	42 (3.4%)	44 (9.1%)		10 (3.5%)	30 (7.0%)		4.5%	6.7%	
Died intubated	14 (1.1%)	10 (2.1%)		4 (1.4%)	9 (2.1%)		1.6%	1.9%	
Acute use of renal replacement therapy	37 (3.0%)	21 (4.4%)	0.15	14 (5%)	18 (4%)	0.599	4.8%	3.5%	0.307
Multisystem organ failure	32 (2.6%)	22 (4.6%)	0.033	16 (5.7%)	19 (4.4%)	0.402	4.9%	3.8%	0.436
Post-operative neurological dysfunction			<0.001			0.013			<0.001
None	1211 (97%)	450 (94%)		273 (97%)	403 (94%)				
Stroke	13 (1.0%)	17 (3.5%)		1 (0.4%)	16 (3.7%)		0.8%	3.5%	
Transient ischaemic attack	19 (1.5%)	14 (2.9%)		8 (2.8%)	12 (2.8%)		1.7%	2.9%	
Pulmonary complications	156 (13%)	107 (22%)	<0.001	49 (17%)	91 (21%)	0.179	16%	19%	0.206
Gastrointestinal complications	42 (3.4%)	24 (5.0%)	0.12	14 (5.0%)	20 (4.6%)	0.829	5%	5%	0.966
Intensive care unit length of stay (days)	1 (1–1)	1 (1–2)	<0.001	1 (1–1)	1 (1–2)	0.022	1 (1–1)	1 (1–2)	0.058
Post-operative length of stay (days)	8 (6–11)	10 (7–16)	<0.001	8 (6–12)	10 (7–15)	<0.001	8 (6–12)	10 (7–15)	<0.001
Discharge destination			<0.001			0.001			<0.001
Convalescence (non-acute hospital)	28 (2.3%)	13 (2.7%)		5 (1.8%)	12 (2.8%)		2.1%	2.9%	
Home	1117 (90%)	363 (76%)		246 (87%)	329 (77%)		87%	77%	
Not applicable—patient deceased	45 (3.6%)	36 (7.5%)		14 (5%)	31 (7.2%)		5.4%	7.1%	
Other acute hospital	52 (4.2%)	67 (14%)		17 (6%)	57 (13%)		5%	12%	
Unknown	1 (0.08%)	2 (0.4%)		0	0		0.2%	0.3%	

Data as median (Q1–Q3); count (%). Genetic matched repair cohort is weighted to account for multiplicity (i.e. use of replacement). Continuous variables compared using Wilcoxon rank sum test for all patients and weighted Wilcoxon rank sum test for matched and overlap weighted patients. Categorical variables compared using Pearson's Chi-squared test for all patients and weighted Pearson's Chi-squared test for matched and overlap weighted patients.

During follow-up, 64 patients required first mitral valve reoperation: 54 (4.3%) in the repair group (5.7 per 1000-patient-years) and 10 (2.1%) in the replacement group (2.4 per 1000-patient-years). Among repair patients, reoperation rates differed by technique: 3/13 patients without annuloplasty required redo compared to 51/1230 with annuloplasty.

**Figure 3:**
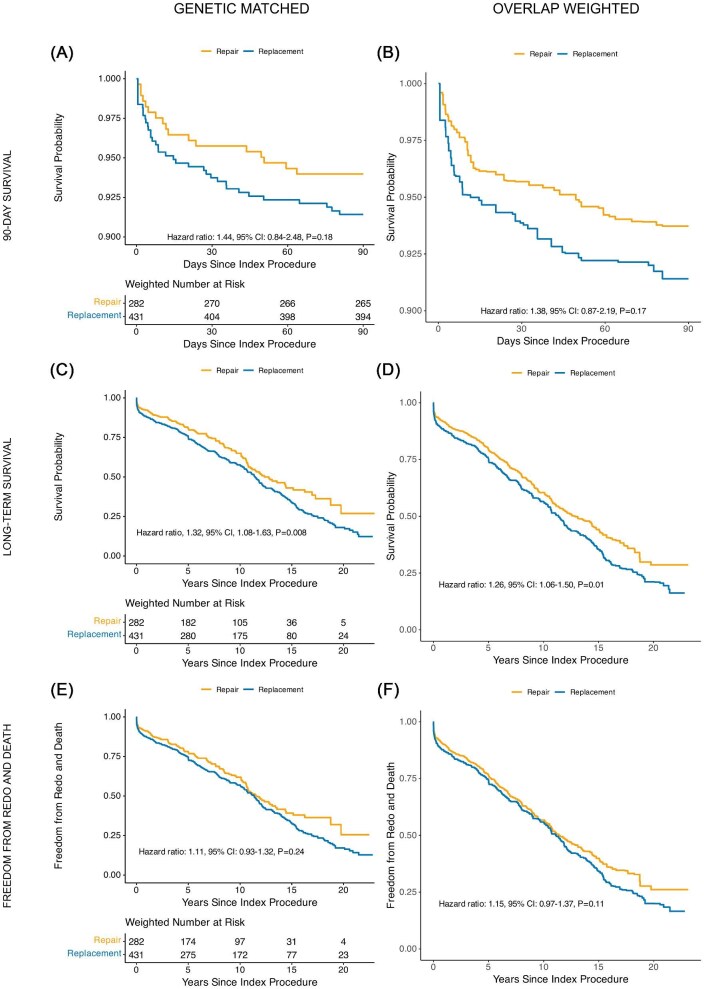
Time-to-event analysis on all mitral regurgitation patients comparing 90-day survival (*A*–*B*), long-term survival (*C*–*D*), and time to redo and death (*E*–*F*) in mitral repair and replacement after covariate balance with genetic matching (panels *A*, *C*, *E*) and overlap weighting (panels *B*, *D*, *F*).

Survival analysis demonstrated no significant difference in 90-day mortality between replacement and repair groups [Hazard ratio (HR): 1.44, 95% CI: 0.84–2.48, *P* = 0.18], consistent in both matched and overlap-weighted analyses (*Figure 3*A–B). However, long-term follow-up showed a significant survival benefit for repair (HR: 1.32, 95% CI: 1.08–1.63, *P* = 0.008) (*Figure 3*C–D). The composite outcome of first reoperation and mortality was not significantly different between groups (HR: 1.11, 95% CI: 0.93–1.32, *P* = 0.24) (*Figure 3*E–F). The proportional hazards assumption was met for all time-to-event analyses, with non-significant global Schoenfeld residual tests.

### Degenerative mitral regurgitation

In the subgroup of patients with degenerative MR, 771 patients underwent mitral valve repair, and 288 underwent replacement (*Table 3*). After genetic matching, baseline characteristics were well balanced between the repair and replacement groups, each consisting of 287 patients, with SMD below 10% (*Table 3*). In the OW cohort, exact covariate balance was achieved.

**Table 3:** Patient characteristics according to mitral valve repair or replacement in the degenerative mitral regurgitation subgroup

	All patients with degenerative MR			Genetic matched			Overlap weighted^a^	
Characteristic	Repair, *N* = 771	Replacement, *N* = 288	SMD	Repair, *N* = 287	Replacement, *N* = 287	SMD	Repair, ESS = 495	Replacement, ESS = 242
Age (years)	67 (12)	68 (11)	0.09	69 (10)	68 (11)	0.06	68 (11)	68 (12)
Female sex	273 (35%)	122 (42%)	0.14	109 (38%)	121 (42%)	0.08	40%	40%
Body mass index (kg/m^2^)	26 (4)	26 (5)	0.04	26 (4)	26 (5)	0.07	26 (4)	26 (5)
Smoking status								
Current Smoker	34 (4%)	13 (4%)	0.01	8 (3%)	13 (5%)	0.08	4%	4%
Ex-smoker	330 (43%)	158 (55%)	0.24	163 (57%)	158 (55%)	0.04	52%	52%
Never smoked	407 (53%)	117 (41%)	0.25	116 (40%)	116 (40%)	0	44%	44%
Chronic pulmonary disease	78 (10%)	39 (14%)	0.11	33 (11%)	39 (14%)	0.06	11%	11%
Pulmonary hypertension	175 (23%)	132 (46%)	0.50	139 (48%)	131 (46%)	0.06	37%	37%
Previous CVA or TIA	66 (9%)	28 (10%)	0.04	21 (7%)	28 (10%)	0.08	9%	9%
Neurological dysfunction	17 (2%)	17 (6%)	0.19	17 (6%)	17 (6%)	0	4%	4%
Diabetes	44 (6%)	24 (8%)	0.1	21 (7%)	24 (8%)	0.04	7%	7%
Creatinine >200 µmol/L	21 (3%)	29 (10%)	0.30	24 (8%)	28 (10%)	0.05	6%	6%
Atrial fibrillation	455 (59%)	173 (60%)	0.02	166 (58%)	173 (60%)	0.05	59%	59%
Arterial hypertension	408 (53%)	154 (53%)	0.01	155 (54%)	154 (54%)	0.01	53%	53%
Previous cardiac surgery	16 (2%)	14 (5%)	0.15	10 (4%)	14 (5%)	0.06	3%	3%
NYHA class			0.32			0.08		
1/2	422 (55%)	112 (39%)		101 (35%)	112 (39%)		45%	45%
3/4	349 (45%)	176 (61%)		186 (65%)	175 (61%)		55%	55%
Left ventricular ejection fraction (LVEF) category								
Poor (LVEF ≤30%)	17 (2%)	15 (5%)	0.16	12 (4%)	14 (5%)	0.03	4%	4%
Moderate (LVEF 31%–50%)	110 (14%)	66 (23%)	0.22	59 (21%)	66 (23%)	0.06	18%	18%
Good (LVEF >50%)	644 (84%)	207 (72%)	0.28	216 (75%)	207 (72%)	0.07	78%	78%
Extracardiac arteriopathy	27 (4%)	29 (10%)	0.26	23 (8%)	29 (10%)	0.07	7%	7%
Myocardial infarction in the last 90 days	16 (2%)	21 (7%)	0.25	24 (8%)	21 (7%)	0.04	5%	5%
Critical pre-operative state	7 (1%)	18 (6%)	0.21	12 (4%)	17 (6%)	0.07	2%	2%
Logistic EuroSCORE	6 (6)	10 (10)	0.41	7 (3)	7 (4)	0.08	8 (7)	8 (8)
Operative urgency								
Elective	704 (91%)	232 (81%)	0.31	238 (83%)	232 (81%)	0.05	87%	87%
Urgent	66 (9%)	44 (15%)	0.21	41 (14%)	44 (15%)	0.03	13%	13%
Emergency or salvage	1 (0.1%)	12 (4%)	0.28	8 (3%)	11 (4%)	0.05	1%	1%
Coronary artery bypass graft	151 (20%)	128 (44%)	0.55	129 (45%)	127 (44%)	0.01	35%	35%
Tricuspid valve surgery	214 (28%)	39 (14%)	0.36	34 (12%)	39 (14%)	0.05	18%	18%

Data as *n* (%) or mean (SD).

a Overlap weights provide exact covariate balance and all SMD are equal to 0.

**Figure 4:**
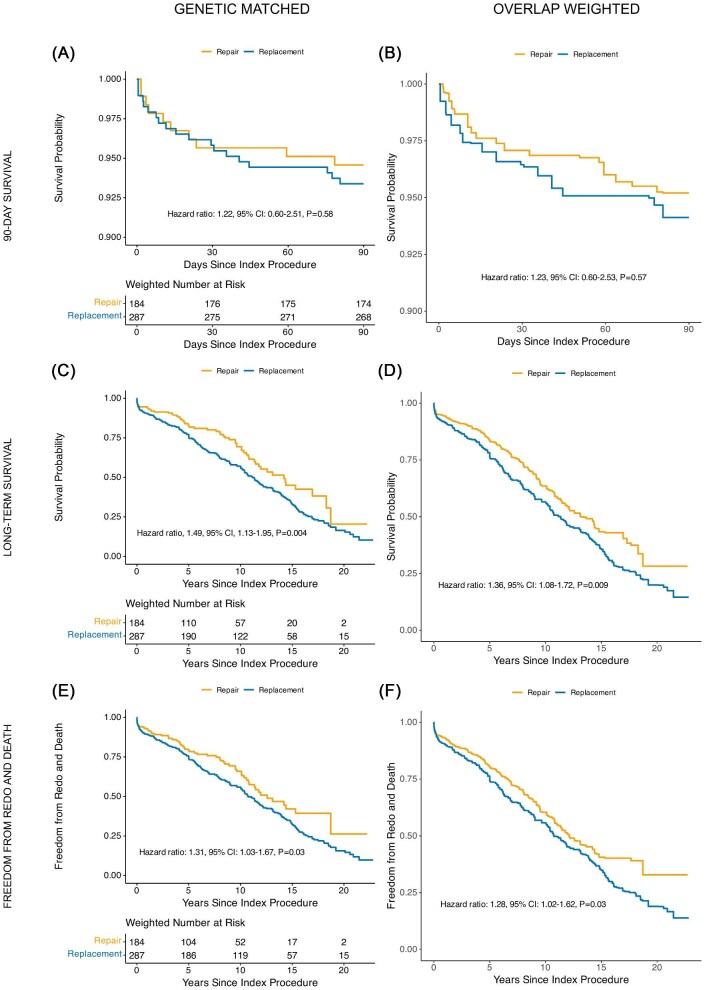
Time-to-event analysis on degenerative mitral regurgitation patients comparing 90-day survival (*A*–*B*), long-term survival (*C*–*D*), and time to redo and death (*E*–*F*) in mitral repair and replacement after covariate balance with genetic matching (panels *A*, *C*, *E*) and overlap weighting (panels *B*, *D*, *F*).

In the genetic-matched cohort and OW cohort, replacement patients had more blood loss, higher transfusion rates, longer hospital stays, and higher rates of neurological complications compared to repair patients (*Table 4*). Genetic-matched and OW cohorts survival analysis showed consistent findings with survival not significantly different at 90 days after surgery in repair and replacement groups (*Figure 4*A–B), whereas long-term survival (*Figure 4*C–D) and composite of redo and death did favour mitral repair over replacement (*Figure 4*E–F).

**Table 4:** Intraoperative and postoperative outcomes among patients with degenerative mitral regurgitation undergoing repair or replacement

	All patients with degenerative MR			Genetic matched (weighted)			Overlap weighted		
Characteristic	Repair, *N* = 771	Replacement, *N* = 288	*P*-value	Repair, *N* = 184	Replacement, *N* = 287	*P*-value	Repair, ESS = 495	Replacement, ESS = 242	*P*-value
Blood loss (mL)	360 (240–580)	460 (300–820)	<0.001	360 (240–600)	460 (300–820)	0.0005	400 (260–640)	440 (269–860)	0.022
Blood used	2 (1–3)	4 (2–6)	0.005	32 (17%)	79 (28%)	0.007	19%	27%	0.027
Transfused blood (units)	0 (0–0)	0 (0–1)	<0.001	0 (0–1%)	0 (0–1)	0.006	0 (0–1)	0 (0–2)	0.022
Cumulative bypass time (min)	148 (124–179)	149 (116–187)	0.8	150 (125–180)	149 (116–187)	0.697	147 (123–180)	146 (113–183)	0.404
Cumulative cross clamp time (min)	105 (85–127)	101 (76–133)	0.3	107 (86–128)	100 (76–134)	0.295	105 (85–127)	100 (76–134)	0.407
Ventilation duration			<0.001			0.106			0.152
Immediate extubation	4	1		17 (9%)	41 (14%)				
<12 h	684 (89%)	224 (78%)		160 (87%)	224 (78%)		87%	80%	
12–24 h	62 (8%)	42 (15%)		17 (9%)	41 (14%)		9%	14%	
>24 h	17 (2%)	16 (6%)		6 (3%)	16 (6%)		3%	4%	
Died intubated	4	5		1	5		1%	1%	
Acute use of renal replacement therapy	15 (2%)	6 (2%)	0.89	6 (3%)	6 (2%)	0.399	3%	2%	0.055
Multisystem organ failure	15 (5%)	25 (9%)	0.08	9 (5%)	5 (2%)	0.044	5%	2%	0.032
Post-operative neurological dysfunction			<0.001			0.008			0.014
None	754 (98%)	266 (92%)		180 (98%)	265 (92%)		98%	92%	
Stroke	2	12 (4%)		1 (0.5%)	12 (4%)		0.6%	4%	
Transient ischaemic attack	15 (2%)	10 (4%)		3 (1.6%)	10 (3%)		2%	4%	
Pulmonary complications	35 (12%)	40 (14%)	0.09	27 (15%)	52 (18%)	0.293	13%	16%	0.21
Gastrointestinal complications	10 (3%)	15 (5%)	0.1	10 (5%)	11 (4%)	0.38	5%	4%	0.339
Intensive care unit length of stay (days)	2 (1–4)	3 (2–5)	0.01	1 (1–1)	1 (1–2)	0.034	1 (1–1)	1 (1–2)	0.126
Post-operative length of stay (days)	7 (5–9)	8 (6–10)	0.03	8 (6–11)	9 (7–14)	<0.001	8 (6–11)	9 (7–14)	<0.001
Discharge destination			<0.001			0.026			0.037
Convalescence (non-acute hospital)	11 (1%)	7 (2%)		2 (1%)	7 (2%)		1.5%	2%	
Home	716 (93%)	229 (80%)		163 (89%)	228 (80%)		90%	83%	
Not applicable—patient deceased	17 (2%)	14 (5%)		9 (5%)	14 (5%)		3.7%	4.0%	
Other acute hospital	26 (3%)	36 (13%)		10 (5%)	36 (13%)		4.5%	10.2%	
Unknown	1	2		0	2		0.3%	0.6%	

Data as median (Q1–Q3); count (%). Genetic matched repair cohort is weighted to account for multiplicity (i.e. use of replacement). Continuous variables compared using Wilcoxon rank sum test for all patients and weighted Wilcoxon rank sum test for matched and overlap weighted patients. Categorical variables compared using Pearson's Chi-squared test for all patients and weighted Pearson's Chi-squared test for matched and overlap weighted patients.

### Ischaemic and functional mitral regurgitation

In the subgroup with ischaemic (*N* = 217) or functional (*N* = 273) MR, 401 patients underwent mitral valve repair (151 ischaemic and 250 functional), and 89 underwent replacement (66 ischaemic and 23 functional) (*Table 5*). After genetic matching, baseline characteristics were balanced between the repair and replacement groups (83 patients each), with SMDs less than 10%. The OW cohort achieved exact covariate balance.

**Table 5:** Patient characteristics according to mitral valve repair or replacement in the ischaemic/functional mitral regurgitation subgroup

	All patients with ischaemic/functional MR			Genetic matched			Overlap weighted^a^	
Characteristic	Repair, *N* = 401	Replacement, *N* = 89	SMD	Repair, *N* = 83	Replacement, *N* = 83	SMD	Repair, ESS = 246	Replacement, ESS = 81
Age (years)	68 (10)	66 (10)	0.17	67 (10)	67 (10)	0.01	67 (10)	67 (10)
Female sex	134 (33%)	36 (40%)	0.15	31 (37%)	34 (41%)	0.04	41%	41%
Body mass index (kg/m^2^)	27 (5)	27 (4)	0.13	27 (4)	27 (4)	0.08	27 (5)	27 (4)
Smoking status								
Current smoker	35 (9%)	15 (17%)	0.35	11 (13%)	11 (13%)	0.00	14%	14%
Ex-smoker	228 (57%)	42 (47%)	0.21	40 (48%)	40 (48%)	0.00	49%	49%
Never smoked	138 (34%)	32 (36%)	0.04	32 (39%)	32 (39%)	0.00	37%	37%
Chronic pulmonary disease	66 (16%)	13 (15%)	0.05	11 (13%)	13 (16%)	0.02	15%	15%
Pulmonary hypertension	83 (21%)	30 (34%)	0.30	25 (30%)	26 (31%)	0.01	28%	28%
Previous CVA or TIA	42 (10%)	4 (4.5%)	0.23	3 (4%)	4 (5%)	0.01	5%	5%
Neurological dysfunction	14 (4%)	5 (6%)	0.10	4 (4.8%)	5 (6%)	0.01	5%	5%
Diabetes	70 (17)	13 (15%)	0.08	11 (13%)	13 (16%)	0.02	16%	16%
Creatinine >200 µmol/L	33 (8.2%)	10 (11%)	0.10	7 (8.4%)	9 (11%)	0.02	10%	10%
Atrial fibrillation	218 (54%)	47 (53%)	0.03	40 (48%)	43 (52%)	0.04	53%	53%
Arterial hypertension	252 (63%)	49 (55%)	0.16	46 (55%)	48 (58%)	0.02	59%	59%
Previous cardiac surgery	16 (4%)	4 (5%)	0.03	2 (2%)	3 (4%)	0.01	4%	4%
NYHA class			0.27			0.02		
1/2	211 (53%)	35 (39%)		32 (39%)	34 (41%)		45%	45%
3/4	190 (47%)	54 (61%)		51 (61%)	49 (59%)		55%	55%
Left ventricular ejection fraction (LVEF) category								
Poor (LVEF ≤30%)	57 (14%)	23 (26%)	0.26	20 (24%)	21 (25%)	0.01	23%	23%
Moderate (LVEF 31%–50%)	139 (35%)	33 (37%)	0.04	35 (42%)	31 (37%)	0.05	37%	37%
Good (LVEF >50%)	205 (51%)	33 (37%)	0.29	28 (34%)	31 (37%)	0.04	40%	40%
Extracardiac arteriopathy	43 (11%)	13 (15%)	0.12	11 (13%)	12 (14%)	0.01	15%	15%
Myocardial infarction in the last 90 days	83 (21%)	27 (30%)	0.22	20 (24%)	22 (27%)	0.02	24%	24%
Critical preoperative state	18 (4.5%)	19 (21%)	0.52	12 (14%)	14 (17%)	0.02	12%	12%
Logistic EuroSCORE	10 (10)	15 (14)	0.43	13 (14)	14 (13)	0.07	9 (10)	9 (10)
Operative urgency								
Elective	300 (75%)	50 (56%)	0.44	52 (63%)	49 (59%)	0.04	65%	65%
Urgent	90 (22%)	25 (28%)	0.13	21 (25%)	24 (29%)	0.04	26%	26%
Emergency or salvage	11 (3%)	14 (16%)	0.37	10 (12%)	10 (12%)	0.00	8%	8%
Coronary artery bypass graft	247 (62%)	69 (78%)	0.35	65 (78%)	63 (76%)	0.02	74%	74%
Tricuspid valve surgery	82 (20%)	13 (15%)	0.15	10 (12%)	13 (16%)	0.04	16%	16%

Data as *n* (%) or mean (SD).

a Overlap weights provide exact covariate balance and all SMD are equal to 0.

**Table 6:** Intraoperative and postoperative outcomes among patients with ischaemic/functional mitral regurgitation undergoing repair or replacement

	All patients with ischaemic/functional MR			Genetic matched (weighted)			Overlap weighted		
Characteristic	Repair, *N* = 401	Replacement, *N* = 89	*P*-value	Repair, *N* = 70	Replacement, *N* = 83	*P*-value	Repair, ESS = 246	Replacement, ESS = 81	*P*-value
Blood loss (mL)	460 (300–720)	470 (300–760)	0.58	470 (340–790)	480 (320–760)	0.72	480 (340–760)	450 (300–700)	0.64
Blood used	103 (26%)	33 (37%)	0.030	24 (34%)	30 (36%)	0.81	29%	34%	0.38
Transfused blood (units)	0 (0–1)	0 (0–2)	0.009	0 (0–2)	0 (0–2)	0.63	0 (0–1)	0 (0–2)	0.25
Cumulative bypass time (min)	156 (133–185)	169 (142–203)	0.021	159 (143–191)	168 (141–203)	0.45	159 (133–188)	168 (140–203)	0.17
Cumulative cross clamp time (min)	107 (84–128)	100 (84–142)	0.84	109 (89–125)	100 (83–144)	0.58	107 (84–127)	102 (84–144)	0.81
Ventilation duration			0.003			0.19			0.59
Immediate extubation	0	0		0	0		0	0	
<12 h	320 (80%)	57 (64%)		51 (73%)	55 (66%)		74%	69%	
12–24 h	49 (12%)	14 (16%)		12 (17%)	12 (14%)		15%	13%	
>24 h	22 (5.5%)	14 (16%)		3 (4%)	12 (14%)		7%	14%	
Died intubated	10 (2.5%)	4 (4.5%)		4 (6%)	4 (5%)		4%	3%	
Acute use of renal replacement therapy	18 (4.5%)	9 (10%)	0.067	4 (6%)	8 (10%)	0.35	7%	9%	0.49
Multisystem organ failure	13 (3%)	9 (10%)	0.009	3 (4%)	9 (11%)	0.11	5%	10%	0.10
Postoperative neurological dysfunction			>0.99			0.98			0.83
None	386 (96%)	86 (97%)		67 (96%)	80 (96%)		96%	97%	
Stroke	11 (3%)	2 (2%)		2 (3%)	2 (2%)		2%	2%	
Transient ischaemic attack	4 (1%)	1 (1%)		1 (2%)	1 (1%)		2%	1%	
Pulmonary complications	73 (18%)	22 (25%)	0.16	15 (21%)	19 (23%)	0.83	3%	5%	0.60
Gastrointestinal complications	14 (4%)	8 (9%)	0.041	3 (4%)	8 (10%)	0.18	4%	9%	0.10
Intensive care unit length of stay (days)	1 (1–1)	1 (1–3)	<0.001	1 (1–2)	1 (1–3)	0.19	1 (1–2)	1 (1–3)	0.15
Postoperative length of stay (days)	8 (6–13)	10 (6–16)	0.10	9 (7–15)	10 (6–16)	0.60	9 (7–14)	10 (6–15)	0.39
Discharge destination			<0.001			0.001			<0.001
Convalescence (non-acute hospital)	15 (4%)	4 (5%)		0	4 (5%)		2%	5%	
Home	338 (84%)	53 (60%)		60 (86%)	49 (59%)		83%	62%	
Not applicable—patient deceased	25 (6%)	15 (17%)		7 (10%)	15 (18%)		9%	16%	
Other acute hospital	23 (6%)	17 (19%)		3 (4%)	15 (18%)		6%	17%	

Data as median (Q1–Q3); count (%). Genetic matched repair cohort is weighted to account for multiplicity (i.e. use of replacement). Continuous variables compared using Wilcoxon rank sum test for all patients and weighted Wilcoxon rank sum test for matched and overlap weighted patients. Categorical variables compared using Pearson's Chi-squared test for all patients and weighted Pearson's Chi-squared test for matched and overlap weighted patients.

In the genetic-matched cohort, differences in intra- and post-operative outcomes between repair and replacement patients were attenuated. No significant differences were observed in blood loss, transfusion requirements, or cumulative bypass times, rates of multisystem organ failure, gastrointestinal complications, or cerebrovascular accidents. Nevertheless, replacement patients were still less likely to be discharged home and more likely to be discharged to other care facilities. Similar findings were observed in OW cohorts.

**Figure 5:**
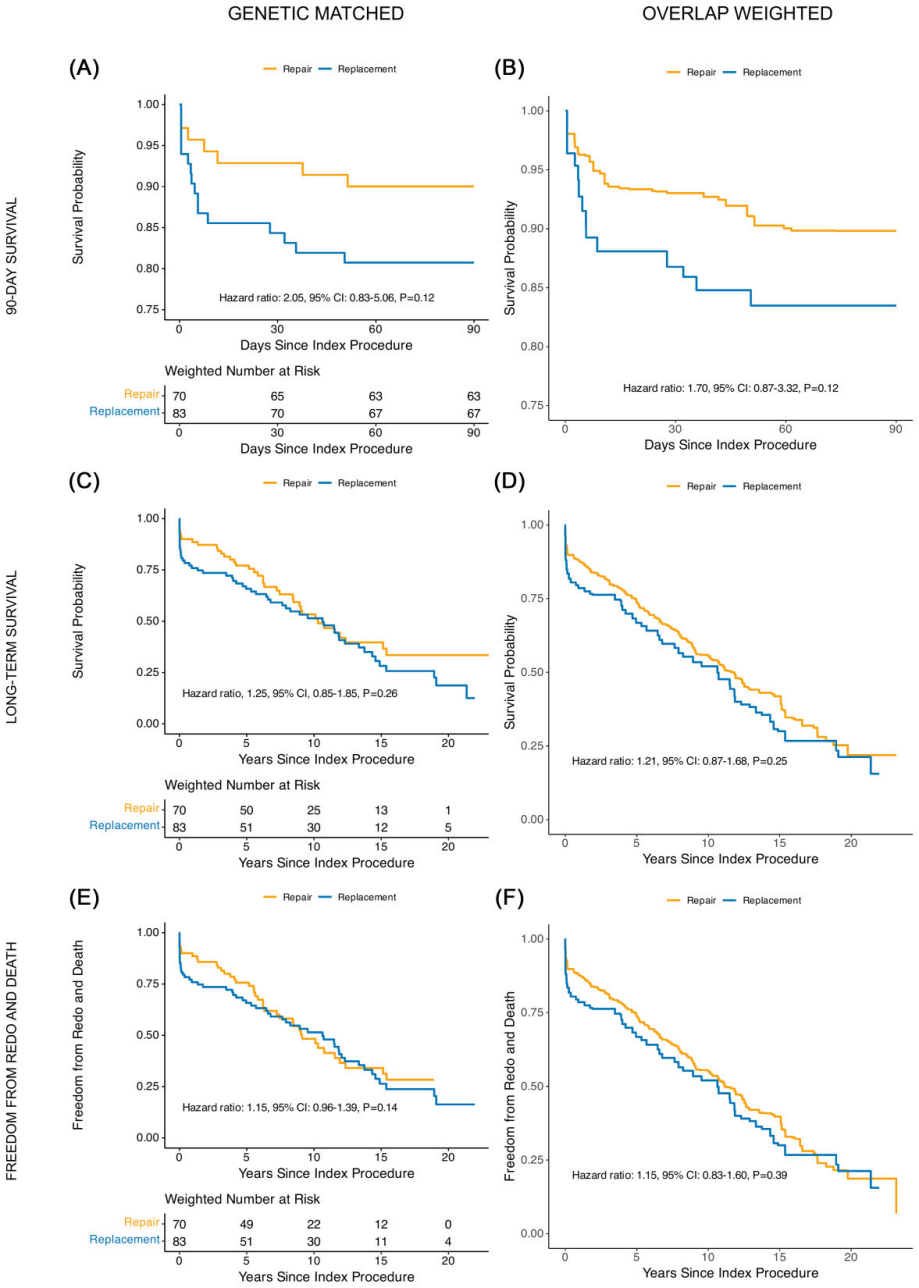
Time-to-event analysis on ischaemic/functional mitral regurgitation patients comparing 90-day survival (*A*–*B*), long-term survival (*C*–*D*), and time to redo and death (*E*–*F*) in mitral repair and replacement after covariate balance with genetic matching (panels *A*, *C*, *E*) and overlap weighting (panels *B*, *D*, *F*).

Genetic-matched and OW cohort survival analysis showed consistent findings with no statistically significant differences in survival at 90 days after surgery (*Figure 5*A–B), long-term survival (*Figure 5*C–D), and composite of redo and death (*Figure 5*E–F) between mitral repair and replacement strategies.

## Discussion

In this large, single-centre, retrospective study, we found that mitral valve repair was associated with a significant increase in long-term survival compared to mitral valve replacement in patients with MR. Although mitral valve repair resulted in a higher rate of reoperation compared to replacement, the composite outcome of reoperation and mortality did not differ significantly between the two groups in both matched and overlap weighted (OW) cohorts. This suggests that while repair may lead to more reoperations, it does not adversely affect overall long-term survival when considering both mortality and the need for reintervention.

The numerous differences in baseline characteristics were consistent with previous literature, where patients undergoing replacement were more unwell, with more symptoms, more complex valvular pathology and left ventricular dysfunction. Therefore, it is important to note that a matched sample of patients with repair has more comorbidities as compared to the general population undergoing mitral valve repair.^19^ In a comprehensive analysis of predictors of replacement vs. repair we found that patients with pulmonary hypertension, concomitant CABG and previous cardiac surgery had increased odds of mitral replacement vs. repair. A key finding of our study was the higher rate of perioperative complications in the replacement group. After matching of baseline characteristics, patients with replacement required more blood transfusion, had more cerebrovascular complications and had longer intensive care and hospital length of stay. There was no difference in gastrointestinal and respiratory complications. These findings contrast with Chikwe *et al.*, who observed more gastrointestinal complications in elderly patients in the replacement group but similar rates of stroke between groups.^20^ Our findings align with a meta-analysis of observational studies which showed higher rates of thromboembolic complications with replacement compared to repair across all mitral valve aetiologies.^21^ This reinforces the recommendation for mitral valve repair in degenerative MR when a durable repair is feasible.

Our subgroup analysis of patients with degenerative MR demonstrated that mitral valve repair was associated with superior long-term survival and reoperation-free survival compared to replacement. Patients undergoing repair had lower rates of intraoperative blood loss, required fewer transfusions, experienced shorter hospital stays, and had fewer neurological complications. These results are consistent with previous observational studies, which have reported better survival and lower morbidity with mitral valve repair in degenerative MR.^22–24^ Our findings can be compared with the MIDA registry, a large multicenter study analysing outcomes in 1922 patients (1709 repairs and 213 replacements) during mean follow-up of 9.2 years.^22^ Twenty-year survival in propensity score matched population was better after MV repair than after MV replacement (41% vs. 24%, *P* < 0.001). MV repair also was associated with a reduced incidence of reoperations and valve-related complications (i.e. stroke, bleeding, and endocarditis). Our study adds to this evidence by demonstrating that the survival benefit of repair persists in a more contemporary cohort, while also highlighting important differences in early complications, particularly the higher risk of stroke and transfusion requirements with replacement.

The operative strategy of ischaemic and functional MR is more controversial.^25^^,^^26^ Our findings in the ischaemic/functional MR subgroup differ from those reported by Deja *et al.*, who observed a significant survival benefit associated with mitral valve repair over replacement in patients with secondary MR.^26^ In their analysis of the Polish National Registry, involving 7633 patients with functional or ischaemic MR, they found that mitral valve replacement was an independent predictor of mortality in multivariable Cox regression analysis (HR: 1.32; 95% CI: 1.17–1.49). This association persisted after propensity score matching, with the replacement group exhibiting higher long-term mortality (HR: 1.24; 95% CI: 1.06–1.45). In contrast, our study did not demonstrate a significant difference in long-term survival or reoperation-free survival between mitral repair and replacement in patients with ischaemic/functional MR after balancing covariates. This discrepancy may be attributed to several factors: their much larger sample size and number of events compared to our analysis, but also shorter follow-up (median 3.4 years) and lower risk cohort (logistic EuroSCORE II 2.83 for repair and 2.75 for replacement). Our findings align more closely with the results of the Cardiothoracic Surgical Trials Network (CTSN) randomized controlled trial, which reported no significant difference in survival at 2 years between repair and replacement for severe ischaemic MR and a higher recurrence rate of MR after repair.^7^ Our study contributes to the ongoing discussion by providing evidence that, in our patient population, matched patients who received either mitral repair or replacement exhibited comparable long-term survival in ischaemic/functional MR.

The main strength of this study is the nearly complete prospectively collected and validated dataset of patients undergoing mitral valve operations from a reference centre with expertise in mitral valve repair techniques and minimally invasive approach.^27^^,^^28^ In addition, median follow-up of over 7 years is one of the longest in the literature. Furthermore, to balance the differences in baseline covariates, we carefully matched patients using a combination of exact and genetic matching and performed a sensitivity analysis using OW. In a recent simulation study, genetic matching with replacement yielded better covariate balance than nearest neighbour matching with calliper, full, and optimal matching algorithms, and subsequent treatment effects were nearly unbiased.^29^

### Limitations

Our trial has several limitations, hence the associations reported in the present analysis should be considered with caution. Clinical outcomes are limited to inpatient morbidity and long-term mitral reoperation if performed in our centre and all-cause mortality captured nationwide from UK's Office for National Statistics. Data on post-operative echocardiographic results or data on post-discharge complications or cause of death were not available. We did not assess surgeon-specific variability in the operative approach. It has been shown that increased surgeon-level mitral volume was independently associated with an increased probability of mitral repair.^30^

The gold-standard way to evaluate the present study hypothesis is a randomized controlled trial (RCT) which can balance both measured and unmeasured confounders between comparison groups by the mechanism of randomization, observational studies usually suffer from confounding effects. However, as it is well known that randomization of patients with suitable anatomy for repair to mitral valve replacement, especially in degenerative MR might be considered unethical, the observational data is the best evidence available to us at present. We used genetic matching and OW to address measured confounding, but one cannot exclude the possibility of unmeasured confounding. Additionally, there was an imbalance in treatment choice, with more patients being offered repair than replacement, particularly in patients with functional MR where only 23 out of 273 received replacement. Therefore, these findings are not generalizable to patients who would have not been considered for replacement.

### Implications

It is apparent that a large scale randomized controlled trial comparing repair and replacement is unlikely to ever be performed, therefore the observational data is the best evidence available for us to draw comparisons between the treatment methods. The present study findings are in line with current guideline recommendations of mitral valve repair rather than replacement if technically feasible. The study also highlights the need to develop strategies limiting intraoperative blood loss and the need for transfusion and improve prevention of cerebrovascular complications in patients undergoing mitral valve replacement.

## Conclusion

We found that mitral valve repair reduced long-term mortality among patients with MR and no previous valve surgery. The survival benefit was most pronounced in degenerative MR, supporting current guidelines favouring repair in this population. However, in secondary MR, no significant survival difference was observed between repair and replacement strategies, suggesting the need for individualized decision-making in these patients.

## Supplementary Material

qcae108_Supplemental_Files

## Data Availability

The data underlying this article will be shared on reasonable request to the corresponding author.
