# Supplementary material for: Comparison of mitral valve repair vs. replacement for mitral valve regurgitation
Source: Eur Heart J Qual Care Clin Outcomes. 2025 Jan 7;11(5):587–603. doi: 10.1093/ehjqcco/qcae108 (PMC12342954; doi:10.1093/ehjqcco/qcae108)
Supplement: qcae108_Supplemental_Files [file qcae108_supplemental_files.zip › Figure 3.pdf]

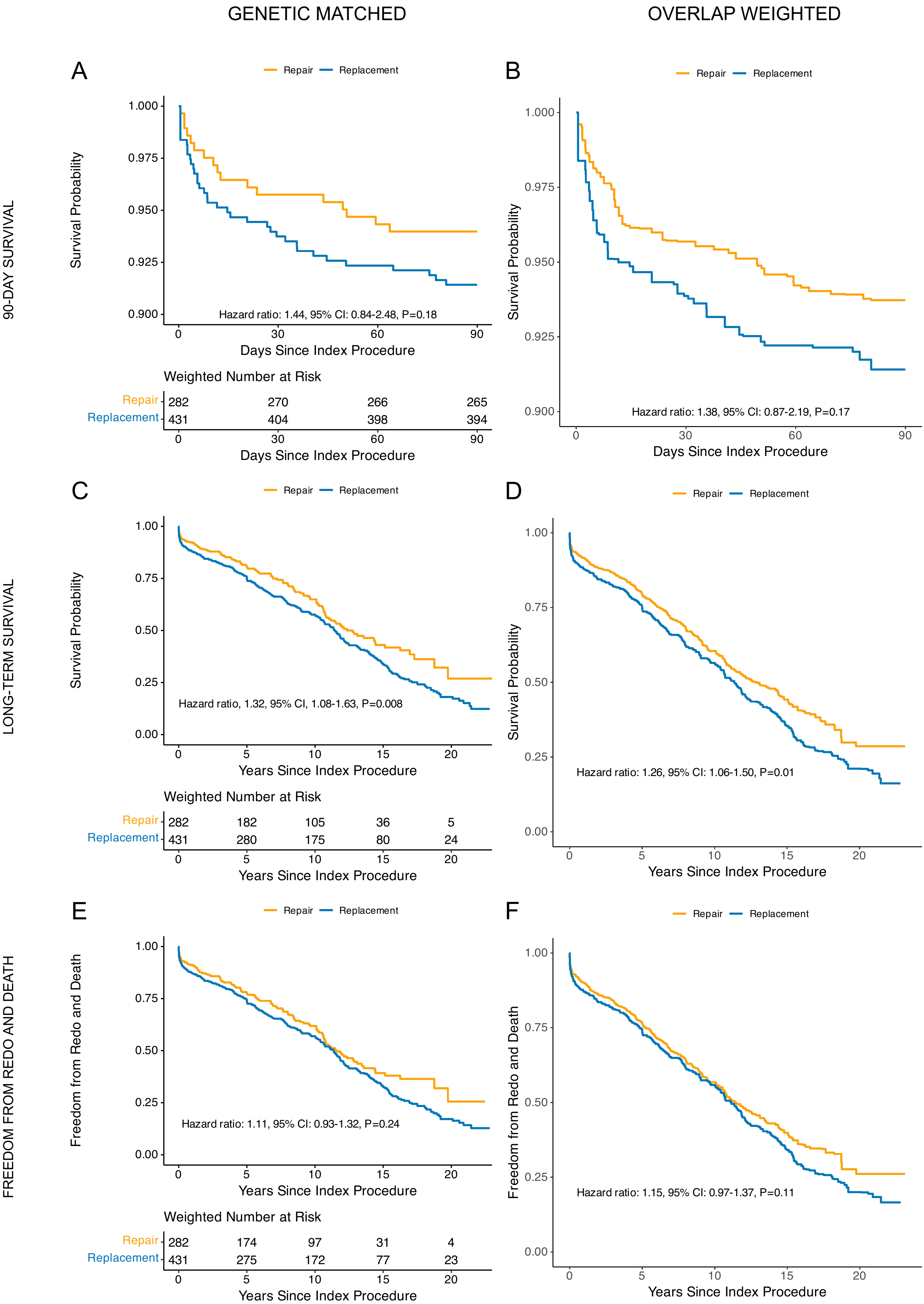
GENETIC MATCHED
OVERLAP WEIGHTED
90-DAY SURVIVAL
LONG-TERM SURVIVAL
FREEDOM FROM REDO AND DEATH
A
Repair
Replacement
Survival Probability
1.000
0.975
0.950
0.925
0.900
Hazard ratio: 1.44, 95% CI: 0.84-2.48, P=0.18
0
30
60
90
Days Since Index Procedure
Weighted Number at Risk
Repair 282 270 266 265
Replacement 431 404 398 394
B
Hazard ratio: 1.38, 95% CI: 0.87-2.19, P=0.17
C
1.00
0.75
0.50
0.25
0.00
Hazard ratio, 1.32, 95% CI, 1.08-1.63, P=0.008
0
5
10
15
20
Years Since Index Procedure
Weighted Number at Risk
Repair 282 182 105 36 5
Replacement 431 280 175 80 24
D
Hazard ratio: 1.26, 95% CI: 1.06-1.50, P=0.01
E
Freedom from Redo and Death
Hazard ratio: 1.11, 95% CI: 0.93-1.32, P=0.24
Weighted Number at Risk
Repair 282 174 97 31 4
Replacement 431 275 172 77 23
F
Hazard ratio: 1.15, 95% CI: 0.97-1.37, P=0.11
