# Supplementary material for: Comparison of mitral valve repair vs. replacement for mitral valve regurgitation
Source: Eur Heart J Qual Care Clin Outcomes. 2025 Jan 7;11(5):587–603. doi: 10.1093/ehjqcco/qcae108 (PMC12342954; doi:10.1093/ehjqcco/qcae108)
Supplement: qcae108_Supplemental_Files [file qcae108_supplemental_files.zip › Figure 4.pdf]

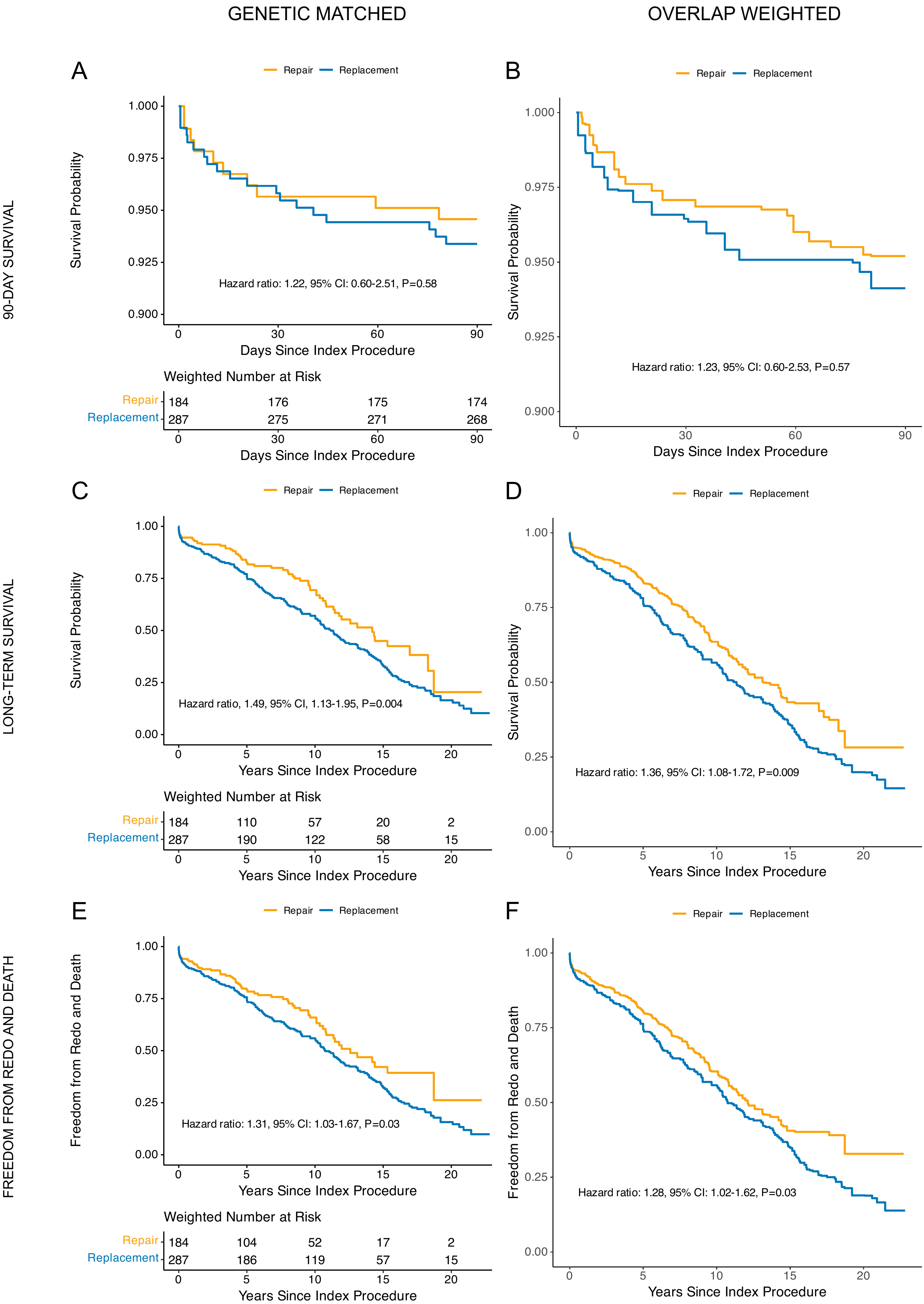
GENETIC MATCHED
OVERLAP WEIGHTED
90-DAY SURVIVAL
LONG-TERM SURVIVAL
FREEDOM FROM REDO AND DEATH
A
Repair
Replacement
Survival Probability
1.000
0.975
0.950
0.925
0.900
Hazard ratio: 1.22, 95% CI: 0.60-2.51, P=0.58
0
30
60
90
Days Since Index Procedure
Weighted Number at Risk
Repair 184 176 175 174
Replacement 287 275 271 268
Days Since Index Procedure
B
Repair
Replacement
Survival Probability
Hazard ratio: 1.23, 95% CI: 0.60-2.53, P=0.57
Days Since Index Procedure
C
Repair
Replacement
Survival Probability
1.00
0.75
0.50
0.25
0.00
Hazard ratio, 1.49, 95% CI, 1.13-1.95, P=0.004
0
5
10
15
20
Years Since Index Procedure
Weighted Number at Risk
Repair 184 110 57 20 2
Replacement 287 190 122 58 15
Years Since Index Procedure
D
Repair
Replacement
Survival Probability
Hazard ratio: 1.36, 95% CI: 1.08-1.72, P=0.009
Years Since Index Procedure
E
Repair
Replacement
Freedom from Redo and Death
Hazard ratio: 1.31, 95% CI: 1.03-1.67, P=0.03
Years Since Index Procedure
Weighted Number at Risk
Repair 184 104 52 17 2
Replacement 287 186 119 57 15
Years Since Index Procedure
F
Repair
Replacement
Freedom from Redo and Death
Hazard ratio: 1.28, 95% CI: 1.02-1.62, P=0.03
Years Since Index Procedure
