# Supplementary material for: Comparison of mitral valve repair vs. replacement for mitral valve regurgitation
Source: Eur Heart J Qual Care Clin Outcomes. 2025 Jan 7;11(5):587–603. doi: 10.1093/ehjqcco/qcae108 (PMC12342954; doi:10.1093/ehjqcco/qcae108)
Supplement: qcae108_Supplemental_Files [file qcae108_supplemental_files.zip › Figure 5.pdf]

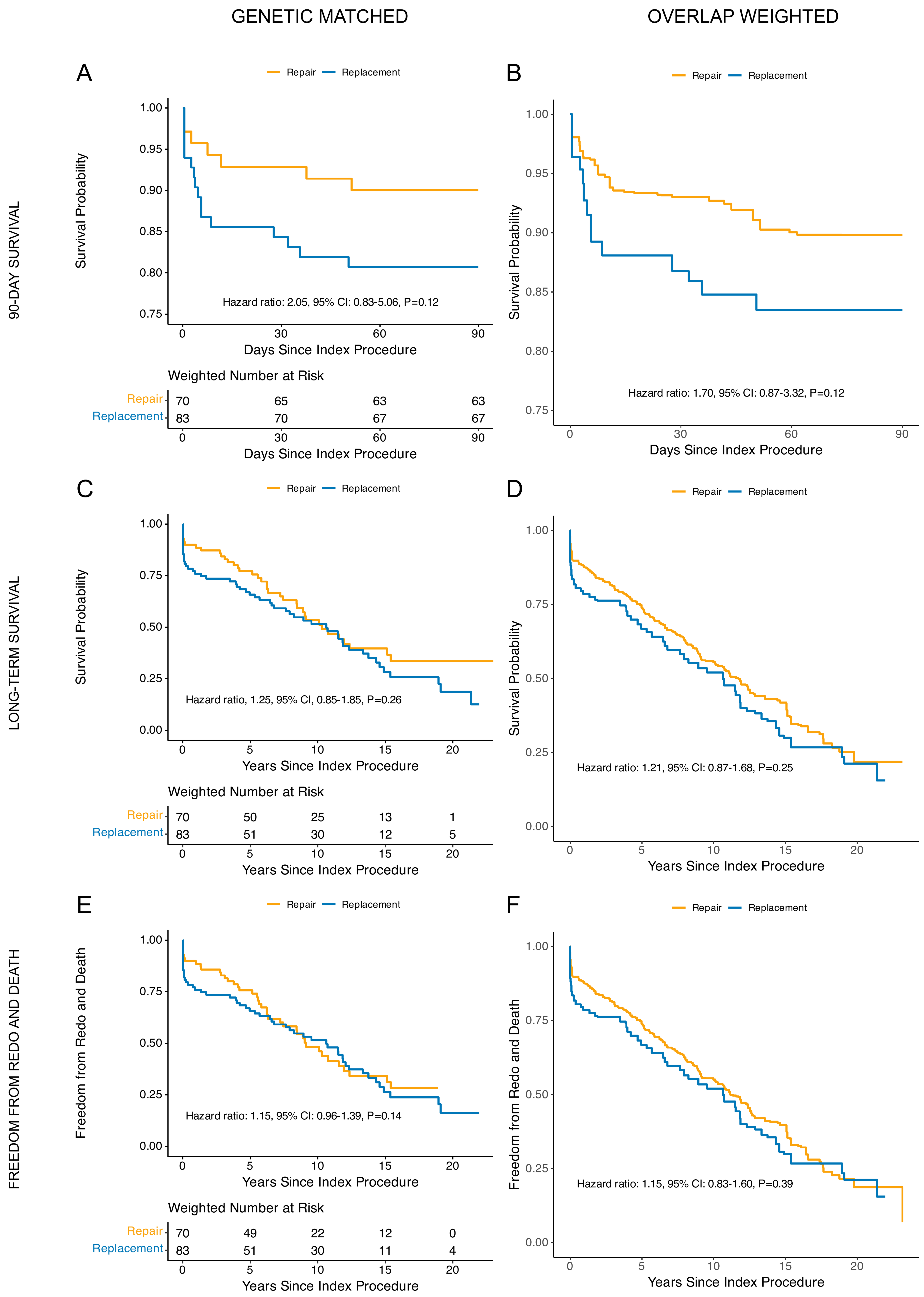

GENETIC MATCHED
OVERLAP WEIGHTED
90-DAY SURVIVAL
LONG-TERM SURVIVAL
FREEDOM FROM REDO AND DEATH
A
Repair
Replacement
Survival Probability
Hazard ratio: 2.05, 95% CI: 0.83-5.06, P=0.12
Days Since Index Procedure
Weighted Number at Risk
Repair 70 65 63 63
Replacement 83 70 67 67
B
Hazard ratio: 1.70, 95% CI: 0.87-3.32, P=0.12
C
Hazard ratio, 1.25, 95% CI, 0.85-1.85, P=0.26
Years Since Index Procedure
Repair 70 50 25 13 1
Replacement 83 51 30 12 5
D
Hazard ratio: 1.21, 95% CI: 0.87-1.68, P=0.25
E
Freedom from Redo and Death
Hazard ratio: 1.15, 95% CI: 0.96-1.39, P=0.14
Repair 70 49 22 12 0
Replacement 83 51 30 11 4
F
Hazard ratio: 1.15, 95% CI: 0.83-1.60, P=0.39
