# Supplementary figures and images for: Comparison of mitral valve repair vs. replacement for mitral valve regurgitation
Source: Eur Heart J Qual Care Clin Outcomes. 2025 Jan 7;11(5):587–603. doi: 10.1093/ehjqcco/qcae108 (PMC12342954; doi:10.1093/ehjqcco/qcae108)

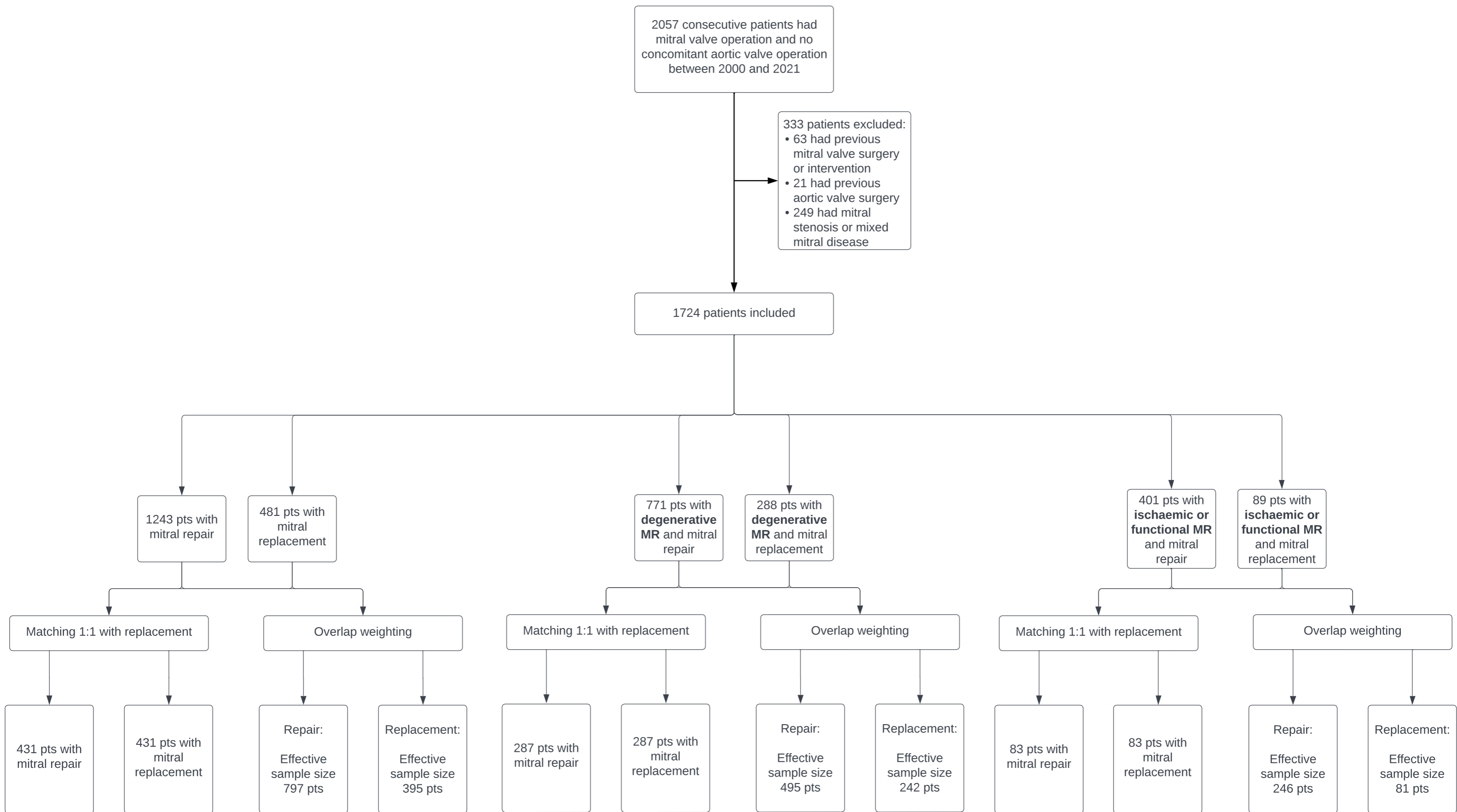

Supplement: qcae108_Supplemental_Files [file qcae108_supplemental_files.zip › Figure 1.pdf]

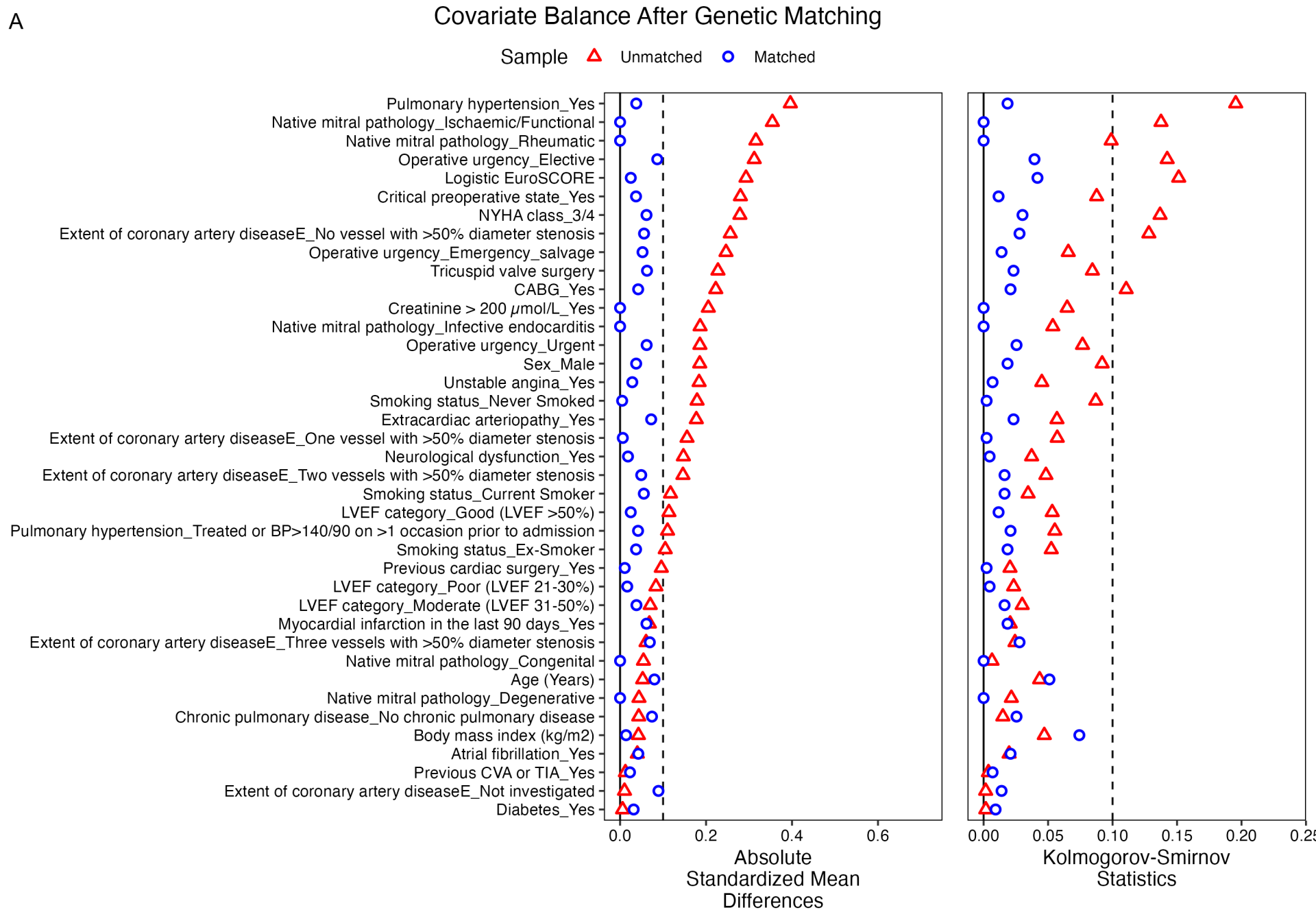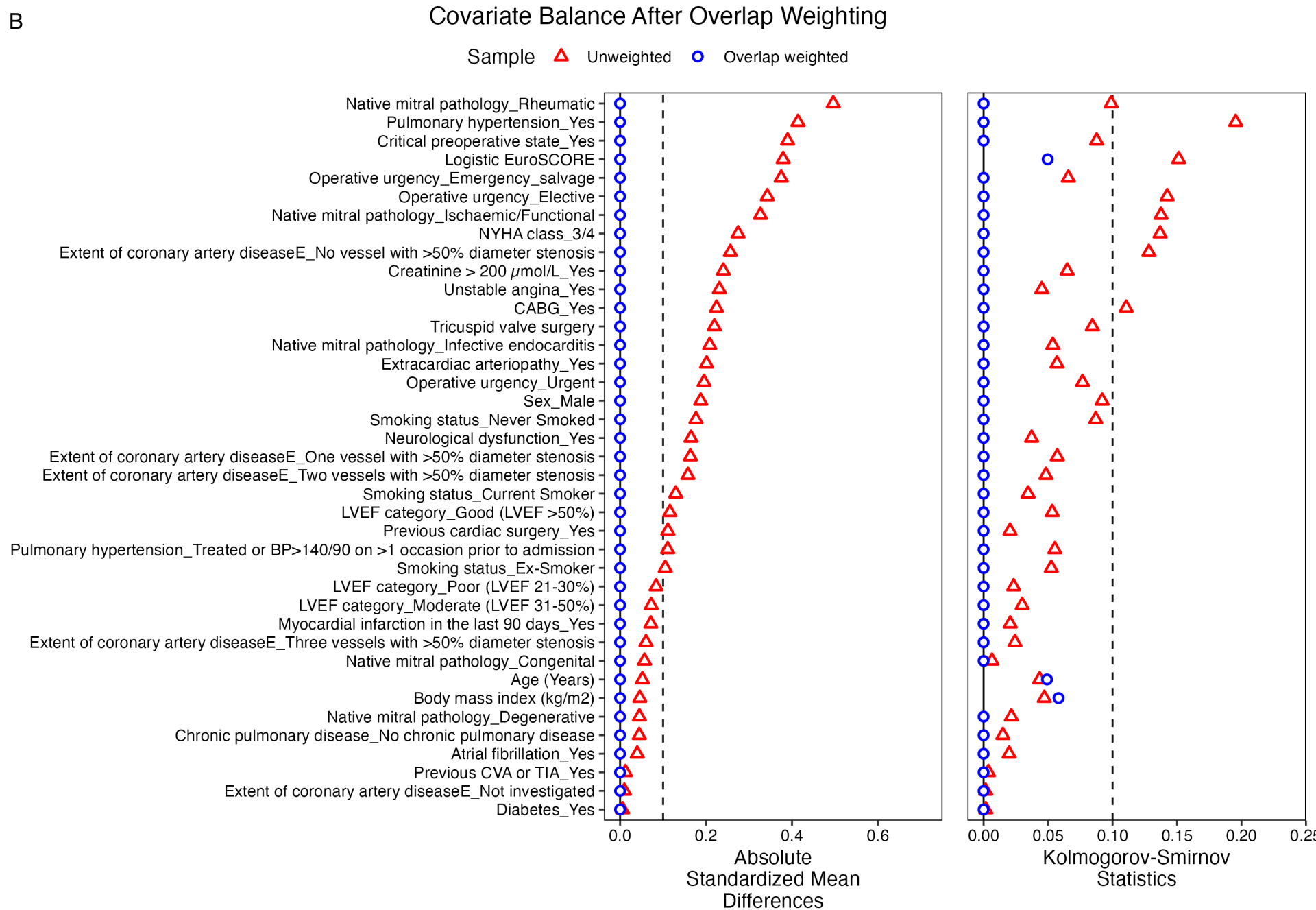

Supplement: qcae108_Supplemental_Files [file qcae108_supplemental_files.zip › Figure 2.pdf]
